# Ligand binding modes from low resolution GPCR models and mutagenesis: chicken bitter taste receptor as a test-case

**DOI:** 10.1038/s41598-017-08344-9

**Published:** 2017-08-15

**Authors:** Antonella Di Pizio, Louisa-Marie Kruetzfeldt, Shira Cheled-Shoval, Wolfgang Meyerhof, Maik Behrens, Masha Y. Niv

**Affiliations:** 1 The Institute of Biochemistry, Food and Nutrition, The Robert H Smith Faculty of Agriculture, Food and Environment, The Hebrew University, 76100 Rehovot, and The Fritz Haber Center for Molecular Dynamics, The Hebrew University, Jerusalem, 91904 Israel; 2 German Institute of Human Nutrition Potsdam-Rehbruecke, Dept. Molecular Genetics, 14558 Nuthetal, Germany; 3 Department of Animal Science, The Robert H. Smith Faculty of Agriculture, Food and Environment, The Hebrew University, Rehovot, 76100 Israel

## Abstract

Bitter taste is one of the basic taste modalities, warning against consuming potential poisons. Bitter compounds activate members of the bitter taste receptor (Tas2r) subfamily of G protein-coupled receptors (GPCRs). The number of functional Tas2rs is species-dependent. Chickens represent an intriguing minimalistic model, because they detect the bitter taste of structurally different molecules with merely three bitter taste receptor subtypes. We investigated the binding modes of several known agonists of a representative chicken bitter taste receptor, ggTas2r1. Because of low sequence similarity between ggTas2r1 and crystallized GPCRs (~10% identity, ~30% similarity at most), the combination of computational approaches with site-directed mutagenesis was used to characterize the agonist-bound conformation of ggTas2r1 binding site between TMs 3, 5, 6 and 7. We found that the ligand interactions with N93 in TM3 and/or N247 in TM5, combined with hydrophobic contacts, are typically involved in agonist recognition. Next, the ggTas2r1 structural model was successfully used to identify three quinine analogues (epiquinidine, ethylhydrocupreine, quinidine) as new ggTas2r1 agonists. The integrated approach validated here may be applicable to additional cases where the sequence identity of the GPCR of interest and the existing experimental structures is low.

## Introduction

Taste is important for the survival of animals, with a small number of taste modalities encoding aversive or attractive reactions to food^1^. Bitter taste is one of the basic taste modalities, thought to protect organisms from consuming poisons that are often bitter. Chemical compounds can be defined as “bitter” if they are verbally described as bitter-tasting by human subjects, or elicit aversive behavior in other animals^2–4^, and activate bitter taste receptors in a selective and dose-responsive manner *in-vitro* ^5^. Bitter taste receptors (Tas2rs) were shown to be expressed also extra-orally, and are emerging as potential novel drug targets^6^. Thus, better understanding of molecular recognition of bitter compounds may be helpful for further elucidation of the physiological roles of these multi-specific, and perhaps pluri-functional, receptors.

Understanding the sense of taste in birds is particularly interesting, since it can provide novel insights into the evolutionary mechanisms of taste perception and food selection, as recently reviewed^7^. Even though avian species typically have low taste bud numbers and few taste receptor genes, genomic, molecular biology and behavioral studies suggest that the avian taste system is well developed and that birds have an accurate capacity to detect different taste modalities^8^. Interestingly, the number of functional Tas2rs is not the only factor determining the importance of bitter taste in a species’ diet, since it was demonstrated that smaller *Tas2r* gene repertoires, as in the case of chicken bitter taste receptors (ggTas2rs), can be compensated by a broader repertoire of ligands^9^.

Tas2rs are seven transmembrane domain (7TM) proteins, usually considered as a subgroup of the Class A G protein-coupled receptors (GPCRs)^10, 11^. The three chicken bitter taste receptor subtypes, ggTas2r1, ggTas2r2 and ggTas2r7, have confirmed expression in the chicken oral cavity and gastrointestinal tract^12, 13^. Here, we study the molecular recognition of bitter compounds by a representative chicken bitter receptor, ggTas2r1. This receptor was found to be functional^13^, ggTas2r1 agonists^9^ and most recently a ggTas2r1 antagonist have been identified^13^. Moreover, in contrast to ggTas2r2 and ggTas2r7, for which orthologous receptors with considerable functional conservation can be found in turkey^9^, ggTas2r1 seems to play a more species-specific role for chicken, as the corresponding turkey receptor has pseudogenized. Hence, among the three chicken Tas2rs, which represent an interesting minimalistic model system for the sense of bitter taste in vertebrates, the ggTas2r1 is the most specific subtype for chickens’ bitter tasting abilities.

Investigation of the molecular recognition between this receptor and its ligands is the first step towards understanding ggTas2r1 activity. Specifically, ligand-receptor complex can shed light on recognition and enable discovery of additional ligands. To date, none of the chemosensory GPCR structures have been solved by X-ray crystallography, and homology modeling tools are required to predict their structures^10, 14^. Modeling GPCRs, including Tas2rs, proved successful in many cases^14–16^, but remains challenging, especially when closely related templates are lacking^17^. To predict the binding mode of known ligands to their GPCR target involves several steps, each requiring appropriate validations^18^. Previous studies on human TAS2Rs found that the binding site of TAS2Rs coincides with the canonical binding site of Class A GPCRs, in the upper third of the extracellularly oriented parts of the 7TM bundle^11, 14, 19–21^. Modeling the residue arrangement of the binding site is of fundamental importance, since it strongly affects the binding mode prediction and the analysis of predicted interactions. Recent studies suggest that binding site optimization may greatly improve model quality and applicability for docking and ligand design: various strategies have been used^22^, such as implementing functional knowledge into the model building process^23^, or introducing residue flexibility in the docking process. The latter can be achieved by applying rigid docking to a large number of different conformations of the receptor^16^, or by applying flexible docking approach to a single model^24, 25^.

**Figure 1:**
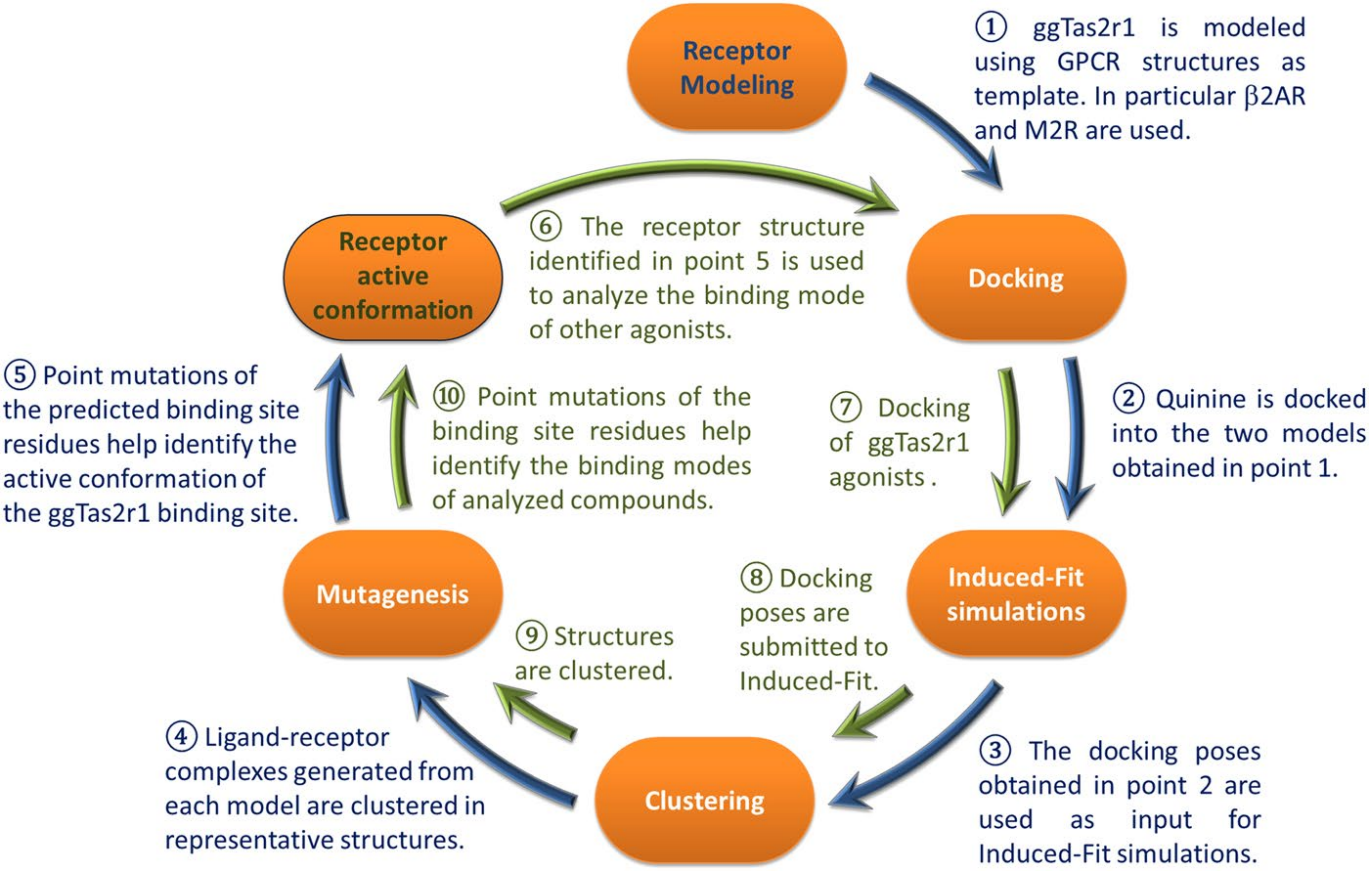
Workflow. The first cycle (blue arrows) led to the investigation of the quinine binding mode and, consequently, the identification of the active conformation of the ggTas2r1 binding site. The second cycle (green arrows) is aimed to identify the binding modes of other ggTas2r1 agonists by using the predicted active conformation. In each cycle, PELE (Protein Energy Landscape Exploration) was used to run Induced-Fit simulations and generate the binding site conformations, while mutagenesis analysis was used to identify and validate the binding modes.

Here, we developed a protocol to identify the binding mode of known ggTas2r1 agonists to their target and to use it for the identification of additional agonists. Specifically, we carried out a flexible docking of quinine to ggTas2r1 model. The binding site arrangement obtained from this investigation was validated by mutagenesis and used to predict the binding modes of six other ggTas2r1 agonists. The binding site flexibility was allowed in order to accommodate the possibility of residue rearrangements for different agonists. Figure 1 shows the workflow used to identify the active conformation of the ggTas2r1 binding site (first cycle, blue arrows), and to subsequently investigate the binding modes of ggTas2r1 agonists (second cycle, green arrows). Finally, the active conformation of the ggTas2r1 binding site was used for a structure-based virtual screening of quinine analogues for which the activity towards ggTas2r1 was not previously known.

## Results

To unravel the binding site conformation of ggTas2r1 we have constructed the structural model, investigated the binding modes of known agonists combining computational and experimental methods, and used the built model to predict new agonists.

### ggTas2r1 structure modeling

Based on previous work^14, 19^, we used single-template homology modeling, in order to allow manual sequence alignment adjustments and direct comparisons of templates. 3D models were built with MEDELLER^26^, specifically developed and validated for template-based modeling of membrane proteins. Two representative agonist-bound GPCR structures in the full active conformation were used as templates: β2 adrenergic receptor β2AR (PDB ID: 3SN6, binding site similarity of 24% to ggTas2r1)^27^ and muscarinic M2 receptor M2R (PDB ID: 4MQS, binding site similarity of 20% to ggTas2r1)^28^ (Fig. 1, step 1). During the project, agonist-bound structures were solved also for μ-opioid (PDB ID: 5C1M^29^) and adenosine A2A (PDB ID: 5G53^30^) receptors. The binding sites of these receptors have even lower sequence similarities to ggTas2r1 binding site (Supplementary Appendix 1) and were not further used here.

Interestingly, β2AR and M2R were solved both in their agonist- and antagonist-bound conformations. The binding site of the β2AR^31^ has modest changes between the two conformations; in contrast, more evident changes are revealed comparing the recent crystal structure of M2R in its active conformation (PDB ID: 4MQS^28^) with the antagonist-bound conformation of the receptor (PDB ID: 3UON)^28, 32^. This underscores the importance of the correct prediction of the binding site conformations. Model 1 and model 2, built from β2AR and M2R, respectively, are shown in Supplementary Figures S1 and S2. SiteMap analysis suggests that the binding site of model 1 is smaller than that of model 2 (Table 1). The residue composition and orientation of the two templates generate different residue arrangements in the models. Consequently, docking simulations of quinine (one of the most potent compounds towards ggTas2r1^9^) into the two models using the same computational settings produce very different results (Fig. 1, step 2). The top scoring docking poses of quinine in model 1 have only hydrophobic interactions vs. hydrophobic interactions and an H-bond interaction between the methoxy oxygen of the quinine with N93 of ggTas2r1 model 2 (docking poses are shown in Supplementary Figure S3).

**Table 1:** Comparison of the binding sites of model 1 and model 2.

Before PELE Simulations	Model 1 Binding site	Model 2 Binding site
H-bond acceptor Area (Å^2^)	82.0	125.5
H-bond donor Area (Å^2^)	272.0	433.5
Hydrophilic Area (Å^2^)	339.4	534.3
Hydrophobic Area (Å^2^)	477.0	502.9
Surface (Å^2^)	937.9	1358.0
**After PELE simulations**		
H-bond acceptor Area (Å^2^)	83.6	74.4
H-bond donor Area (Å^2^)	298.5	244.8
Hydrophilic Area (Å^2^)	370.8	315.8
Hydrophobic Area (Å^2^)	502.1	486.2
Surface (Å^2^)	1051.2	986.9

Because mostly hydrophobic interactions are captured by the models and because of the different results obtained using the two models, binding site optimization is needed. We therefore used a flexible structure-based approach allowing for flexibility of binding site residues during docking (Fig. 1, step 3): PELE (Protein Energy Landscape Exploration) server (https://pele.bsc.es/pele.wt), a stochastic method of mapping large conformational rearrangements and Induced-Fit events in protein-ligand interactions^33–36^. We used the “Ligand binding refinement” protocol available in PELE to include flexibility of the ggTas2r1 binding site residues in complex with quinine. These simulations produce almost 5000 binding poses for each model, allowing an extended analysis of the possible binding modes of quinine in the ggTas2r1 receptor. The structures were clustered into 7 representative structures for model 1, and 5 for model 2 (Fig. 1, step 4).

The quinine molecule reaches the same location in all representative complexes. Examples showing how the binding pocket changes after refinement in each model are shown in Supplementary Figure S4. The ligand-binding refinement leads to a slight increase in the binding site of model 1 and a slight decrease in that of model 2, resulting in similar sizes and residue arrangements of refined binding site. Consequently, the calculated SiteMap descriptors are very similar among the poses generated by the two models. SiteMap descriptors before PELE simulations and average values of 7 poses’ SiteMap descriptors (descriptors for single representative poses are found in Supplementary Tables S1 and S2) after PELE simulations are reported for each model in Table 1.

**Figure 2:**
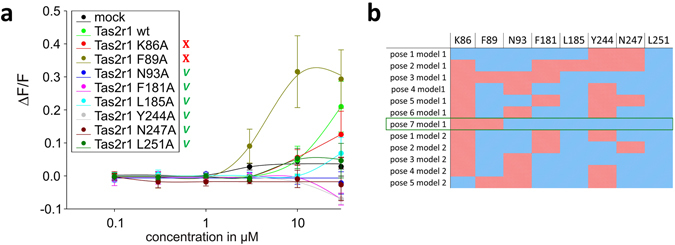
(**a**) Dose-response relations for quinine in cells expressing point-mutated and native ggTas2r1 constructs. The y-axis shows the relative fluorescence changes (ΔF/F), and the x axis the ligand concentration in μM. Green checks indicate residues involved in the binding, red crosses residues that are not involved in the binding, based on experimental data. (**b**) Involvement of residues K86, F89, N93, F181, L185, Y244, N247, and L251 in binding modes of quinine in the representative poses obtained with PELE simulations: the cell is colored in blue if the residue is found to interact with the ligand and in red if there is no interaction. The predicted agonist-bound conformation, pose 7 model 1, is marked by a green frame.

Examination of the poses suggests that residues K86, F89, N93, F181, L185, Y244, N247, and L251, corresponding to Ballesteros-Weinstein (BW)^47^ numbers 3.29, 3.32, 3.36, 5.38, 5.42, 6.47, 6.51, 6.55, respectively, are involved in ligand interactions, though the specific combination of interactions varies: particularly, we checked the possibility for each residue to establish either H-bond or hydrophobic interaction with quinine (Fig. 2).

To identify the pose that optimally describes the quinine/ggTas2r1 interaction, each of the residues K86, F89, N93, F181, L185, Y244, N247, and L251 was mutated to alanine (Fig. 1, step 5). The resulting constructs were transiently transfected in HEK 293T cells stably expressing the chimeric G protein Gα16gust44, and subjected to calcium-mobilization assays using different concentrations of quinine sulfate. Dose-response relationships were obtained and used to evaluate the impact of the individual receptor positions on activation by quinine. Residues, for which the mutation to alanine impairs activation by quinine, are considered to be involved in ligand binding.

The results for K86A and F89A mutants, which show similar or, in case of F89A, improved efficacy properties compared to wild-type ggTas2r1 (Fig. 2a), suggest that there are no interactions between quinine and these residues. Indeed, the strong increase in sensitivity and maximum amplitudes observed for the receptor mutant ggTas2r1_F89A_ indicates a potential negative sterical influence of the wild-type residue at this position on quinine interaction. This excludes the majority of the models: only poses 3 and 7 of model 1 do not implicate favorable interactions of quinine with both of these residues. In contrast to the ggTas2r1_K86A_ and ggTas2r1_F89A_ mutants, mutations at receptor positions 93, 181, 185, 244, 247, and 251 exerted differences in quinine responsiveness as evident from the reduced response magnitudes seen in Fig. 2b. In fact, the ggTas2r1_N93A_, -_F181A_, -_Y244A_, and -_N247A_ mutants resulted in a complete loss of quinine responsiveness, whereas ggTas2r1_L185A_ and -_L251A_ mutants retained residual responses at high quinine concentrations. Pose 7 of model 1 is compatible with all these results, since it has favorable interactions with N93, N247, F181, Y244, L251 and L185 and no observed interactions with K86 and F89 (Fig. 2).

**Figure 3:**
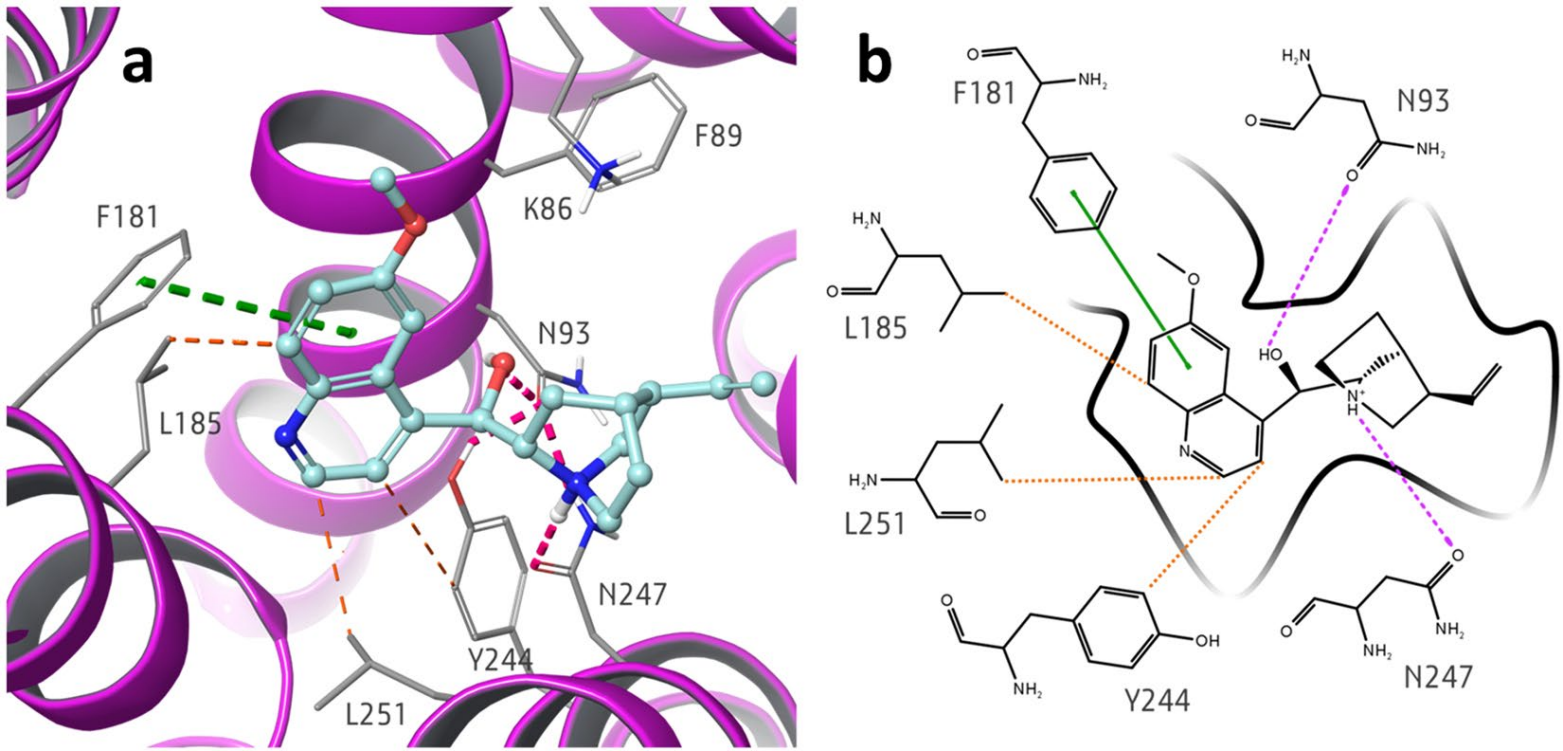
(**a**) 3D and (**b**) 2D representation of the predicted binding mode of quinine at ggTas2r1 receptor. H-bond interactions are shown as magenta dotted lines, π−π interactions as green dashed lines and hydrophobic interactions are represented as the distances between the closest heavy atoms of the ligand and the residue in orange dashed lines.

Specifically, in this pose, quinine establishes H-bonds with N93 and N247, π−π stacking interaction with F181, and hydrophobic interactions with Y244 (distance ligand/Y244: 4.5 Å), L251 (distance of 3.6 Å) and L185 (distance of 3.7 Å) (Fig. 3). Reported distances are between the closest heavy atoms of the receptor and the ligand, unless stated otherwise. The H-bonds between the hydroxyl group of the ligand and N93 carbonyl, and between the ammonium group of the ligand and N247 carbonyl, further extend the intra-receptor Y244-N93-N247 H-bond network.

Thus, pose 7 of model 1 is the working model of the agonist-bound conformation. Next we use it to dock and predict the binding modes of other ggTas2r1 agonists that were shown to activate ggTas2r1 in Behrens *et al*. 2014^9^.

### ggTas2r1 agonists and their binding modes

The ligands (diphenhydramine, chlorpheniramine, diphenidol, chloramphenicol, chloroquine, and coumarin) were tested on the ggTas2r1 mutants introduced above, and their binding modes were explored by docking and PELE simulations starting from pose 7 of model 1 (Fig. 1, steps 6–8, green arrows) in more concise steps (see more details in Methods), since the model was already built and validated in the first cycle with quinine (Fig. 1, steps 1–5, blue arrows).

**Figure 4:**
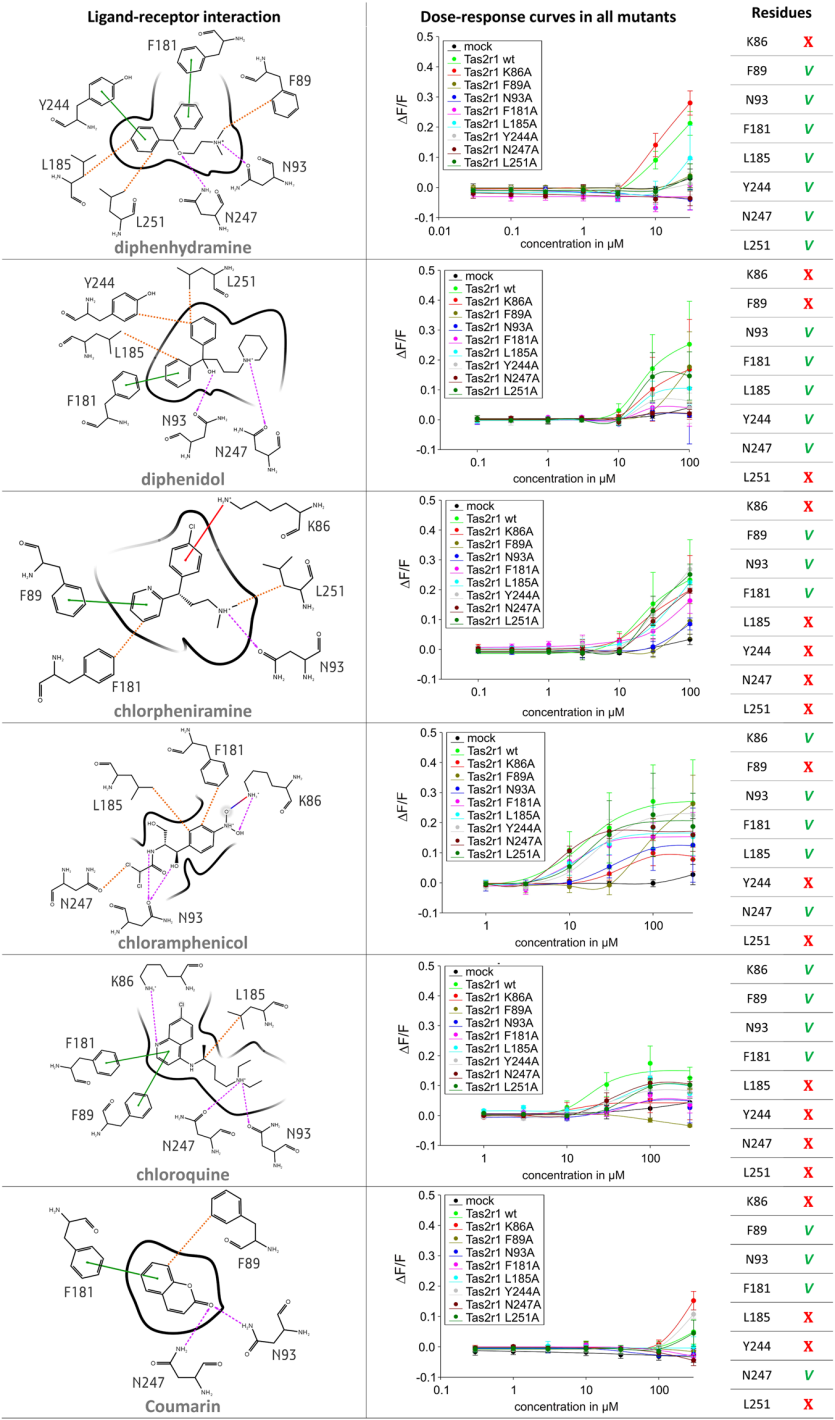
2D representation of predicted binding modes, mutagenesis data and residues involved in the binding of ggTas2r1 agonists. H-bond interactions are shown as magenta dashed arrows, salt bridges as blue-red lines, π−π interactions as green lines, cation−π interactions as red lines and hydrophobic interactions are represented as the distances between the closest heavy atoms of the ligand and the residue as orange dotted lines. Dose-response relations for the specified compounds in cells expressing point-mutated and native ggTas2r1 constructs: the y-axis shows the relative fluorescence changes (ΔF/F), and the x-axis the ligand concentration in μM. Green checks indicate residues involved in the binding, red crosses residues that are not involved in the binding, as deduced from experimental data: we consider “not involved in the binding” those residues that when mutated to alanine show a curve equal (or higher) than that of the wild-type, and “involved” the residues that mutated to alanine have a decreased response (with a change of at least ~0.1 ΔF/F).

For each ligand, the pose in best agreement with functional data was identified by clustering and visual inspection (Fig. 1, steps 9 and 10) (see Fig. 4 and Methods for details).

- Diphenhydramine: The dose-response curves for all mutated receptor constructs except the construct ggTas2r1_K86A_ showed severely decreased signal amplitudes (ggTas2r1_L185A_) or complete lack of activation (ggTas2r1_F89A_, -_N93A_, -_F181A_, -_Y244A_, -_N247A_, -_L251A_) suggesting that all positions except position 86 contribute to diphenhydramine interaction. In the chosen pose, diphenhydramine establishes H-bond interactions with N93 and N247, π-π stacking interactions with F181 and Y244, hydrophobic interactions with F89 (distance of 3.6 Å) through the methyl group, and with L185 (distance of 3.8 Å) and L251 (distance of 3.9 Å) through the aromatic rings.
- Diphenidol: All tested mutants showed variable decrease in responses compared to the wild-type ggTas2r1 upon stimulation with diphenidol. Whereas ggTas2r1_N93A_, -_F181A_, -_Y244A_, and –_N247A_ exhibited severely decreased responses, the constructs ggTas2r1_K86A_, -_F89A_, and -_L251A_ retained considerable activities. In fact, the consequences of the mutation at position 251 seem to be limited to a reduction of the maximal response at the highest diphenidol concentration, whereas the sensitivity of the construct ggTas2r1_L251A_ appears largely unaffected. Interestingly, the construct ggTas2r1_F89A_ is only activated by a high concentration of diphenidol, however, the maximal signal amplitude at 100 µM diphenidol reaches almost that of the wild-type receptor. The most compatible docking pose of diphenidol forms H-bonds with N93 and N247, π-π stacking interaction with F181, and hydrophobic interactions with L185 (distance of 3.6 Å), Y244 (distance of 3.9 Å), and with F89 L251 (distance of 3.9 Å).
- Chlorpheniramine: The functional data for receptor constructs stimulated with chlorpheniramine ranges from wild-type like receptor (ggTas2r1_L185A_ and –_L251A_) to severely impaired (ggTas2r1_N93A_ and –_F89A_). In the compatible docking pose of chlorpheniramine, the ligand establishes an H-bond only with N93 and can form π-π stacking interactions with F89. The hydrophobic interactions are less important: F181 is not in a good orientation for π-π interaction, but can still establish good hydrophobic contacts with the ligand (distance of 4.7 Å); L251 is close to the methyl group of the ligand, but this interaction can be maintained also with a shorter residue such as alanine, since the interaction is established by the Cβ of the leucine (at a distance of 3.7 Å).
- Chloramphenicol: The most severely affected receptor responses upon chloramphenicol application were seen for the constructs ggTas2r1_K86A_ and -_N93A_, with clear shifts of the dose-response towards higher concentrations as well as reduced maximal signal amplitudes. Another set of receptor mutants (ggTas2r1_F181A_, -_L185A_ and -_N247A_) seem to retain full chloramphenicol sensitivity, while signal amplitudes do not reach the levels observed for the native receptor. Few mutants (ggTas2r1_Y244A_ and -_L251A_) show dose-response relations similar to the wild-type receptor. Intriguingly, the construct ggTas2r1_F89A_ shows, similar to what has been observed for diphenidol, a strongly impaired sensitivity, but at high chloramphenicol concentration the maximal signal amplitude is comparable to the native receptor. Chloramphenicol, in its most compatible docking pose with ggTas2r1, forms a salt bridge with K86 and an H-bond with N93. F181 (at a distance of 3.9 Å), L185 (distance of 3.6 Å), and also F89 (distance of 3.4 Å) may allow hydrophobic interactions with the ligand.
- Chloroquine: While the mutated constructs ggTas2r1_L185A_, -_Y244A_, -_N247A_, and -_L251A_ show a mild reduction in maximal response amplitudes compared to the native receptor upon stimulation with chloroquine, the constructs ggTas2r1_K86A_, -_F89A_, -_N93A_, and –_F181A_ show more severe impairments of their activation properties. Regardless of the signal amplitudes, all constructs exhibit reduced sensitivity for chloroquine as evident from the right-shifted dose-response curves. In fact, in the most compatible docking pose, chloroquine binds K86 N93, but also N247, through H-bonds; F89 and F181 through π-π stacking interactions. L251 and Y244 are distant from the ligand (distances of 5.3 Å and 7.0 Å, respectively) but L185 may interact with the ligand via hydrophobic interactions (distance of 3.4 Å).
- Coumarin: The small compound coumarin is only a weak agonist for ggTas2r1. Whereas coumarin-activation persisted for the receptor constructs ggTas2r1_K86A_, -_Y244A_, and –_L251A_, the responses of all other tested constructs were hardly visible. In the most compatible docking pose, coumarin forms H-bonds with both N93 and N247, π-π stacking interaction with F181, and hydrophobic interactions with F89 (distance of 3.7 Å).

The comparison of the compatible binding modes of the known ggTas2r1 agonists analyzed here suggests that the interaction with N93 and/or N247 is present in all complexes. Hydrophobic and aromatic interactions are fundamental as well, but are ligand-dependent, varying mainly according to the ligand size: the hydrophobic contribution to the binding of smaller molecules, as coumarin, is lower.

### Structure-based virtual screening of quinine analogues at ggTas2r1

**Figure 5:**
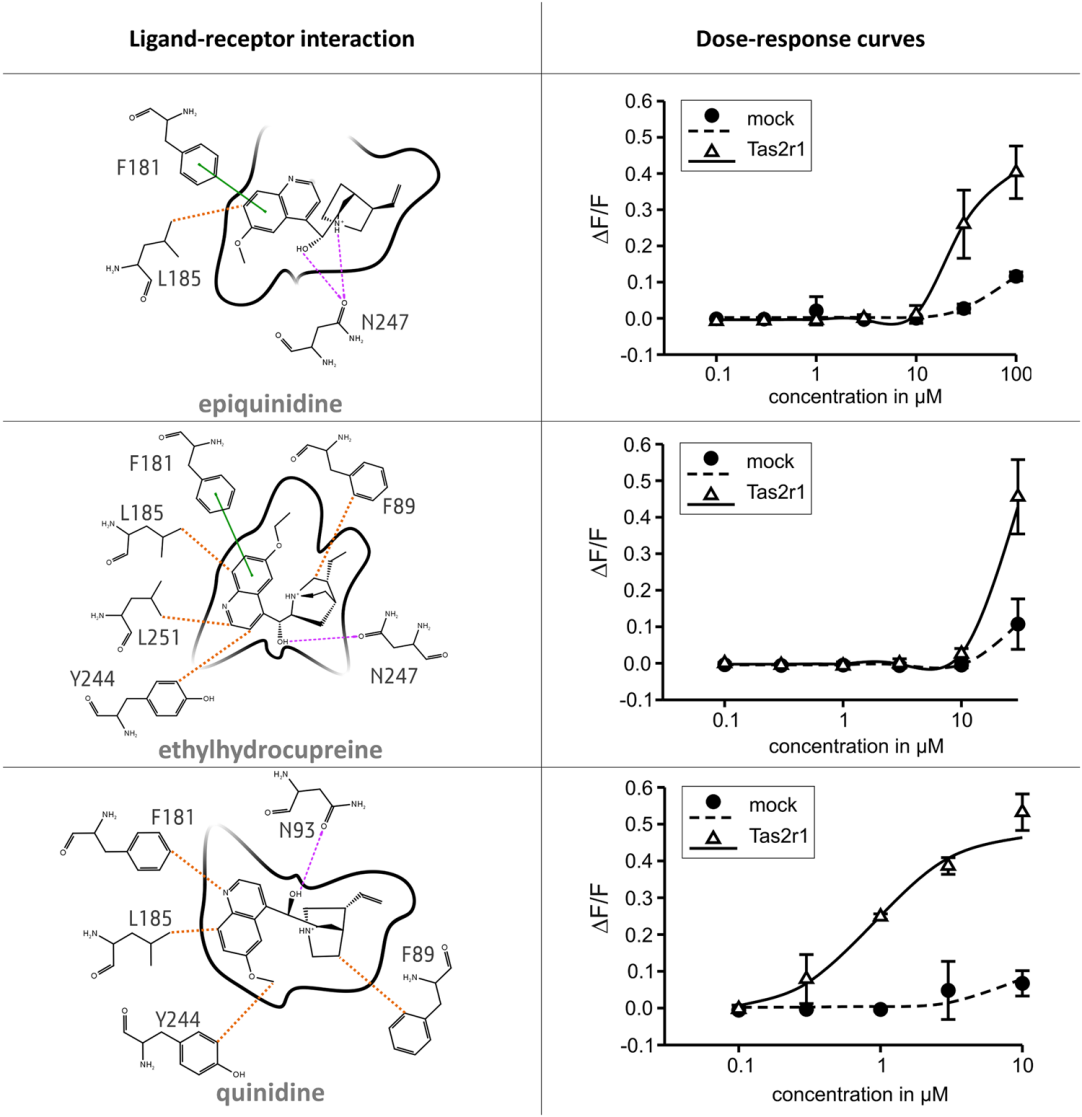
Newly discovered ggTas2r1 agonists, their predicted binding modes and functional data. In the 2D ligand-receptor interaction diagram, H-bond interactions are shown as magenta dashed arrows, salt bridges as blue-red lines, π−π interactions as green lines, cation−π interactions as red lines and hydrophobic interactions are represented as the distances between the closest heavy atoms of the ligand and the residue in orange dotted lines. Dose-response relations for the specified compounds in cells expressing native ggTas2r1: the y-axis shows the relative fluorescence changes (ΔF/F), and the x-axis the ligand concentration in μM.

The analyses performed so far suggest a single model (pose 7 of model 1) that correctly captures the interactions with various known ligands. Can this model be used to predict previously unknown ligands? To answer this question, 62 derivatives of quinine were virtually screened by docking to the ggTas2r1 model. Glide SP score was used as an approximation of binding affinity. Though the docking score neglects entropic contributions, water molecules and uncertainty in loop conformation, it has been successful in capturing the main ligand-receptor interactions and useful in comparing binding modes^37, 38^. Therefore, the docking scores of quinine analogues have been compared to that of quinine and three molecules with scores similar or higher than quinine were selected for *in-vitro* testing: Glide scores of epiquinidine/ggTas2r1, ethylhydrocupreine/ggTas2r1, and quinidine/ggTas2r1 are of −7.882 kcal/mol, −7.408 kcal/mol, and −7.979 kcal/mol, respectively, vs. quinine/ggTas2r1 Glide score of −7.238 kcal/mol. Docking poses and experimental dose-response curves of molecules with the favorable docking scores are reported in Fig. 5.

- Epiquinidine establishes two H-bond interactions with N247 carbonyl through the ammonium and the hydroxyl groups, π-π stacking interaction with F181 and hydrophobic interactions with L185 (distance of 3.1 Å).
- Ethylhydrocupreine establishes an H-bond interaction with N247 carbonyl through the hydroxyl group, π-π stacking interaction with F181 and hydrophobic interactions with F89 (distance of 3.9 Å), L185 (distance of 3.4 Å), Y244 (distance of 4.2 Å), L251 (distance of 3.4 Å).
- Quinidine establishes an H-bond with N93 carbonyl through the hydroxyl group and hydrophobic interactions with F89 (distance of 3.3 Å), F181 (distance of 3.7 Å), L185 (distance of 3.4 Å), Y244 (distance of 3.6 Å).

The response of ggTas2r1 to the three computationally predicted bitter compounds epiquinidine, ethylhydrocupreine and quinidine was experimentally confirmed by calcium-imaging assay. Thus, three new ggTas2r1 agonists were identified (Fig. 5). The most potent of these agonists is quinidine, with a threshold concentration of about 0.3 µM, whereas epiquinidine and ethylhydrocupreine activate the receptor at or beyond 10 µM.

Quinine analogue (8α,9S)-6′-Methoxycinchonan-9-amine trihydrochloride was chosen as a negative control, because it had a less favorable Glide docking score of −6.326 kcal/mol. Indeed, this compound did not activate ggTas2r1 in the artefact-free concentration range (up to 30 µM).

## Discussion

The agonist-bound conformation of the ggTas2r1 was analyzed to identify the binding modes of structurally different bitter compounds in the orthosteric pocket and used to predict additional ggTas2r1 agonists. Since no previous information was available for ggTas2r1 binding site and its agonist-bound conformation, two ggTas2r1 models were generated using two different templates (β2AR and M2R). The presence of the ligand during protein refinement is a well-known strategy for binding site optimization^39^. In the current study, PELE Induced-Fit was used to allow for receptor flexibility during docking of quinine in ggTas2r1 receptor. This approach led to the identification of an agonist-bound conformation of the binding site in agreement with experimental data. This final conformation is different from the initial models, suggesting that sampling residue conformations during docking is essential for optimal binding site prediction.

Comparing all analyzed complexes, we found that hydrophobic interactions and H-bond(s) with N93 and/or N247 are essential for ggTas2r1 activation. Another interesting observation relates to the F89 residue: in the case of quinine binding, the mutation F89A seems even more favorable compared to the wild-type receptor, while in other complexes this mutation reduces the agonist activity. This may be due to the fact that the quinine molecule points to the F89 with its methoxy oxygen while in other ligands, such as diphenidol and chloramphenicol, this location is occupied by hydrophobic groups, while chlorpheniramine establishes π-π stacking interaction with F89. Interestingly, the same residue conformation is predicted by flexible docking of the different ligands, even though the ligand position slightly varies and affects the interaction with this residue (see Supplementary Figure S5). F89 conformation changes only in the coumarin/ggTas2r1 complex, most likely because of the smaller size of the ligand: the residue tends to move inside the binding pocket closer to the ligand. Supplementary Figure S5 shows the superimposition of all described complexes, highlighting that the binding conformations are similar in all the complexes.

The knowledge of the binding site architecture of the ggTas2r1 allowed for the structure-based prediction of previously unknown agonists for this receptor. Quinine analogues were screened by docking and tested in cells, and three new ggTas2r1 agonists (epiquinidine, ethylhydrocupreine and quinidine) were identified. Therefore, the ggTas2r1 model may be considered reliable and predictive.

Our results demonstrate that the combination of *in-silico* and *in-vitro* techniques allows for the rationalization of ligand receptor interactions and the prediction of novel agonists based on the established homology model. This may enable virtual screening of food related activators of chicken bitter taste receptors with implications for the discovery of potentially relevant substances in ecological and agricultural settings.

The current case study provides a generalizable docking strategy for other GPCRs, where the sequence identity between models and templates is very low.

## Methods

### Molecular Modeling

#### ggTas2r1 structure prediction

ggTas2r1 amino-acid sequence was aligned to human TAS2Rs, β2AR and M2R sequences, as previously^14, 40^, using the webserver MAFFT (option*–add* is used to align a full length sequence to a multi sequence alignment)^41^. ggTas2r1 has 31% sequence similarity (9% identity) with M2R and 34% sequence similarity (11% identity) with β2AR. Homology modeling was performed with MEDELLER^26^. β2AR and M2R X-ray structures, PDB IDs: 3SN6^27^ and 4MQS^28^, have been used as templates for model 1 and model 2, respectively. ECL2 was modeled using as template the ELC2 of human kappa opioid receptor since among crystallized GPCRs it has the most similar sequence length with ggTas2r1, does not enter the binding site and does not influence the binding directly, reducing the potential mistakes as a consequence to incorrect loop modeling. The loop was manually inserted in the two models. Side chain refinement and minimization of the models were performed with Prime (version 4.2, Schrödinger, LLC, New York, NY, 2015)^42, 43^, exploiting the option available in this tool which allows to consider the implicit membrane during the calculation. Hydrogen atoms and titratable side chains were optimized with the Protein Preparation Wizard tool from Schrödinger at physiological pH. Ramachandran plots were generated to verify the reliability of the backbone dihedral angles of amino acid residues in the models. Hydrophobic/hydrophilic surfaces were calculated with Maestro (version 10.4, Schrödinger, LLC, New York, NY, 2015) tool using a box margin of 6 Å, confirming that the hydrophobic surface of the TM residues in both model 1 and model 2 highlight the predicted membrane location (Supplementary Figure S1). Binding pockets of the two models were analyzed with SiteMap (version 3.7, Schrödinger, LLC, New York, NY, 2015): protein binding site was defined as the region within 10 Å from residue N93, thus the ECL2 region was excluded and the surface differences were due only to different conformations of residues in TM helices. A fine grid (grid spacing of 0.35 Å) was set to calculate at least 30 site points per site.

#### Docking of quinine to model 1 and model 2

Docking calculations were performed with Glide software (version 6.9, Schrödinger, LLC, New York, NY, 2015). The grids of the models were built with Receptor Grid Generation, setting the box at 10 Å from the residue N93. Quinine was docked into the receptor models using the Standard Precision (SP) accuracy level available in Glide. The xyz coordinates of the top scored poses were used as input for PELE simulations of quinine/model 1 and quinine/model 2 complexes.

#### PELE calculations of quinine/model 1 and quinine/model 2

The “Ligand binding refinement” protocol available in PELE webserver was used for investigating the orientation and conformation of quinine and binding site residues in the quinine/model 1 and quinine/model 2 complexes. 30 parallel 24 hours Monte Carlo simulations were used for sampling. Only the following parameters were modified: energy params ionic was set at 0.15 and caconst at 0.8. 4790 and 5250 structures were obtained from simulations of quinine/model 1 and quinine/model 2, respectively. These structures were clustered using the clustering script available in Schrödinger, based on the RMSD calculated on the ligand atoms and residue atoms 4 Å around them, excluding non-polar hydrogen atoms. Average linkage method of clustering was used. The clustering reduced the poses to 242 for model 1 and 277 for model 2, which were then analyzed and reduced to 92 and 97 for model 1 and 2, respectively, by visual inspections. The clustering procedure was repeated on these selected structures: the final number of clusters is 7 with 0.7 R^2^, clustering strain 1.020, for model 1; and 5 with 0.8 R^2^, clustering strain 1.007, for model 2. The binding sites of the representative structures were analyzed with SiteMap (version 3.7, Schrödinger, LLC, New York, NY, 2015), using the settings described above. Residues defining the binding sites were selected for site-directed mutagenesis.

#### Binding mode predictions of other ggTas2r1 agonists

Diphenhydramin, diphenidol, chlorpheniramin, chloroquin, chloramphenicol, and coumarin were analyzed as ggTas2r1 agonists. The 3D molecular structures of these compounds were downloaded from the publicly accessible electronic database, chicken BitterDB^40^ (http://bitterdb/dbbitter.php?mode_organism=Chicken), and prepared (through the generation of stereoisomers and protonation states at pH 7 ± 0.5) with LigPrep (version 3.6, Schrödinger, LLC, New York, NY, 2015). Pose 7 model 1 resulted in agreement with mutagenesis studies and was used to predict the binding modes of other ggTas2r1 agonists.

Using docking settings described above, the grid of ggTas2r1 pose 7 model 1 structure was prepared and the ligands were docked into it. Generated docking poses were submitted to 10 parallel 24 hours of PELE simulations. 1299 poses were generated for diphenhydramine/ggTas2r1, 1939 for chlorpheniramine/ggTas2r1, 581 for diphenidol/ggTas2r1, 884 for chloroquine/ggTas2r1, 1733 for chloramphenicol/ggTas2r1, and 3324 for coumarin/ggTas2r1. Ligand-receptor complex structures were clustered (using the procedure described for quinine). Representative structures for each ligand were analyzed and binding modes were selected according to the mutagenesis data.

#### Virtual screening of quinine analogues

Structures of 62 quinine analogues (molecules with Tanimoto coefficient between 1.0 and 0.70 based on MOLPRINT 2D fingerprints towards quinine) were obtained from the Sigma-Aldrich catalogue (http://www.sigmaaldrich.com/). The structures were manually built in Maestro using the Built Facility (version 10.4, Schrödinger, LLC, New York, NY, 2015) and prepared with LigPrep (version 3.6, Schrödinger, LLC, New York, NY, 2015) with the same settings used for known ggTas2r1 agonists (see above). Docking-based virtual screening of the compounds was performed with Glide SP (version 6.9, Schrödinger, LLC, New York, NY, 2015) on the pose 7 model 1 ggTas2r1 conformation. The box of the grid was set at 10 Å from the quinine position. Van der Waals scaling of ligands and receptors was set to 0.8.

### Site-directed mutagenesis

*In-vitro* mutagenesis by PCR-mediated recombination^44^ was performed as detailed before^19, 20, 45^ using the cDNA of *ggTas2r1* cloned in the vector pcDNA5/FRT (Invitrogen) available from previous experiments^9^ as template. Briefly, template cDNA was amplified in two separate reactions using CMV forward and the corresponding reverse mutagenesis primer and BGH reverse and the corresponding forward mutagenesis primer, respectively. Next, the two generated subfragments were fused in the following PCR reaction carried out in the presence of CMV forward and BGH reverse primers. The mutated *ggTas2r1* coding region was ligated after EcoR I and NotI restriction with the vector pcDNA5/FRT, which has been modified to result in the in-frame addition of a 5′ sst3-tag and a 3′ hsv-tag^9^. For a list of oligonucleotides used for mutagenesis see Table 2.

**Table 2:** Oligonucleotides used for site-directed mutagenesis of ggTas2r1.

Oligonucleotide	5′ → 3′ Nucleotide sequence
chTas2r1K86A_for	GTAACTTTC**GC** **G**ACAGTTTTTATATTC
chTas2r1K86A_rev	GAATATAAAAACTGT**C** **GC**GAAAGTTAC
chTas2r1F89A_for	CAATGACAGTT**GC** **T**ATATTCCTG
chTas2r1F89A_rev	CAGGAATAT**A** **GC**AACTGTCTTG
chTas2r1N93A_for	GTTTTTATATTCCTG**GC** **C**TCTTATAGC
chTas2r1N93A_rev	GCTATAAGA**G** **GC**CAGGAATATAAAAAC
chTas2r1F181A_for	CAATTTGATT**GC** **T**TTGATCCTTC
chTas2r1F181A_rev	GAAGGATCAA**A** **GC**AATCAAATTG
chTas2r1L185A_for	GATCCTT**GC** **C**TGTAATGTTG
chTas2r1L185A_rev	CAACATTACA**G** **GC**AAGGATC
chTas2r1Y244A_for	CTCCTCCTG**GC** **C**ATTACAAATTTTATC
chTas2r1Y244A_rev	GATAAAATTTGTAAT**G** **GC**CAGGAGGAG
chTas2r1N247A_for	GTACATTACA**GC** **T**TTTATCGCTTTG
chTas2r1N247A_rev	CAAAGCGATAAA**A** **GC**TGTAATGTAC
chTas2r1L251A_for	CAAATTTTATCGCT**GC** **G**ATTCTCATTTTATC
chTas2r1L251A_rev	GATAAAATGAGAAT**C** **GC**AGCGATAAAATTTG
CMV_for	CGCAAATGGGCGGTAGGCGTG
BGH_rev	TAGAAGGCACAGTCGAGG

### Calcium-mobilization assays

The point-mutated expression constructs, the native ggTas2r1 construct, as well as empty expression vectors as controls were transiently transfected in HEK 293T cells stably expressing the chimeric Gα protein Gα16gust44, which were grown in 96-well plates exactly as described previously^9^. About 24 h after transfection, the cells were loaded with the calcium-sensitive dye Fluo4-AM in the presence of 2.5 mM probenecid, washed and placed in a fluorometric imaging plate reader (FLIPR-tetra, Molecular devices). Different concentrations of the bitter compounds (Sigma-Aldrich, Taufkirchen) were automatically applied and changes in fluorescence were monitored. A second application of 100 nM SST-14 stimulating endogenous somatostatin receptors was included as vitality control. Dose-response relations were calculated with SigmaPlot as before.

### Data availability statement

All data generated or analyzed during this study are included in this published article and its Supplementary Information files. Moreover, all mutagenesis data will be made available via GPCRdb^46^ http://gpcrdb.org/ and in the next update of BitterDB http://bitterdb.agri.huji.ac.il/.

## Electronic supplementary material

Supplementary Information
