# Supplementary material for: Ligand binding modes from low resolution GPCR models and mutagenesis: chicken bitter taste receptor as a test-case
Source: Sci Rep. 2017 Aug 15;7:8223. doi: 10.1038/s41598-017-08344-9 (PMC5557796; doi:10.1038/s41598-017-08344-9)
Supplement: Supplementary file 1 — Supplementary Information [file 41598_2017_8344_MOESM1_ESM.pdf]

Supplementary Information

Ligand binding modes from low resolution GPCR models and mutagenesis: chicken bitter taste receptor as a test-case

Antonella Di Pizio, Louisa-Marie Kruetzfeldt, Shira Cheled-Shoval, Wolfgang Meyerhof, Maik Behrens and Masha Y. Niv

Supplementary Figure S1: ggTas2r1 homology models and their templates. Hydrophobic (orange) and hydrophilic (light cyan) surfaces of β2AR crystal structure (A), ggTas2r1 model 1 (B), M2R crystal structure (C), and ggTas2r1 model 2 (D).

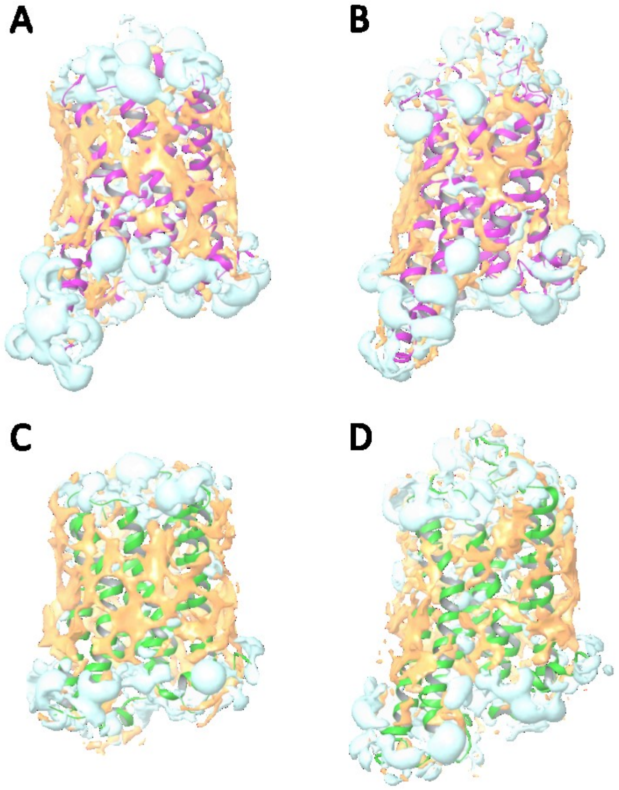

Supplementary Figure S2: ggTas2r1 homology models. ggTas2r1 model 1 (A) is colored in magenta, ggTas2r1 model 2 (B) in green. Binding pocket surfaces of model 1 and model 2 are highlighted in yellow.

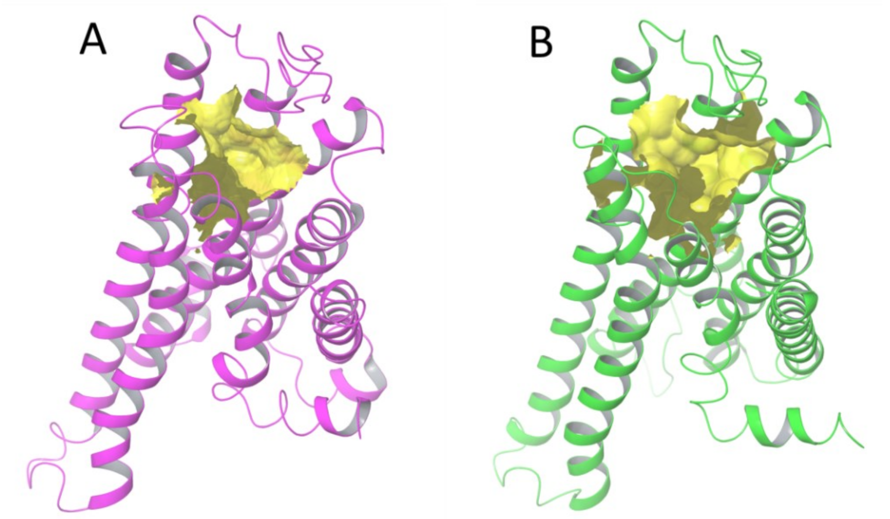

Supplementary Figure S3: a) Docking poses of quinine/model 1 (magenta) **and b) quinine/model 2** (green). H-bond interactions are shown as green dotted lines.

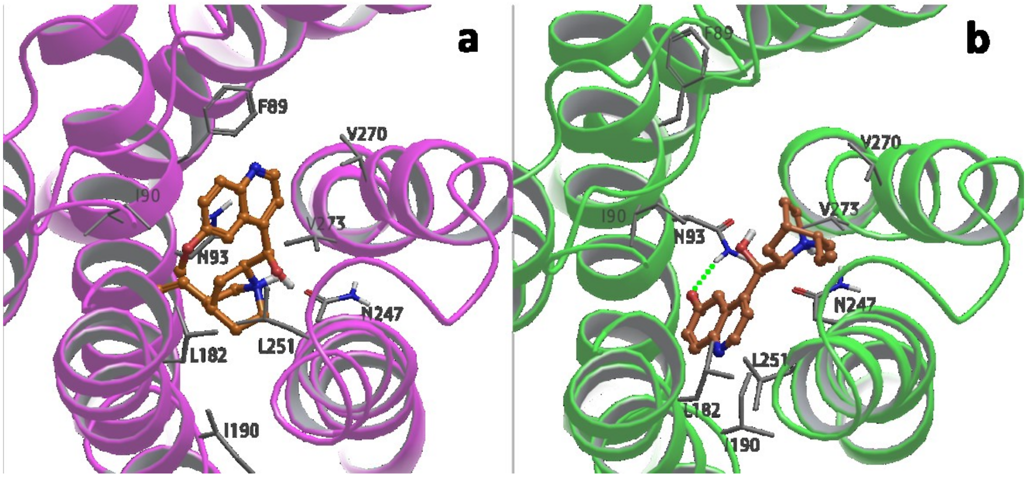

Supplementary Figure S4: Superimposition of quinine/model 1 and quinine/model 2 binding modes obtained after PELE simulations. In the picture we show the representative structures obtained after clustering. Model 1 (magenta) and model 2 (green) receptor structures are shown as transparent cartoon. Panels a and b show the binding modes of two representative quinine/model 1 and quinine/model 2 complexes after PELE simulations superimposed with those of unrefined quinine/model 1 and quinine/model 2 complexes (in black).

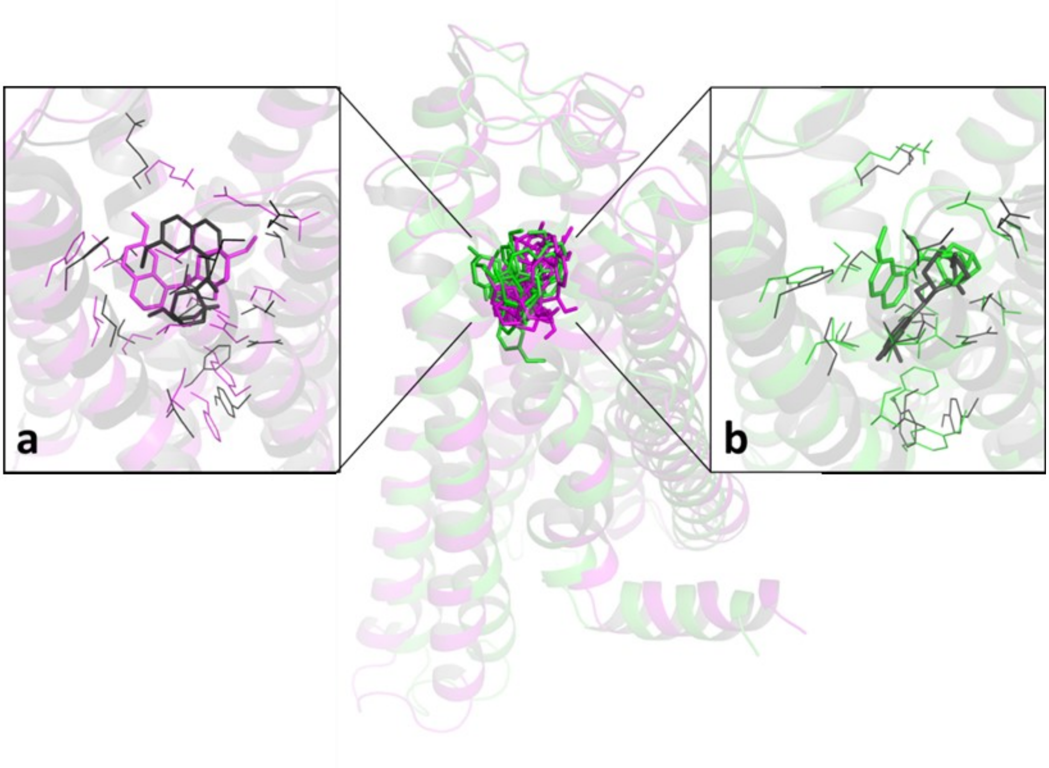

Supplementary Figure S5: Superimposition of all analyzed complexes of ggTas2r1 with its agonists: quinine (magenta), diphenidramine (yellow), diphenidol (cyan), chlorpheniramine (violet), chloramphenicol (orange), chloroquine (green), coumarin (red).

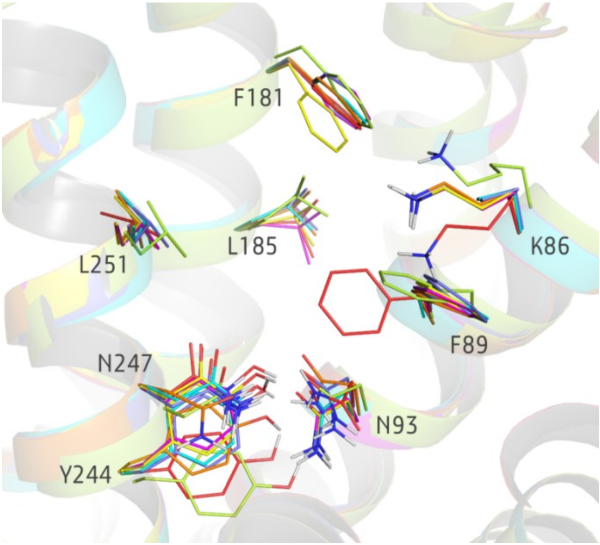

Supplementary Table S1: SiteMap descriptors calculated for ggTas2r1 model1

**MODEL 1**	H-bond acceptor Area (Å^2)	H-bond donor Area (Å^2)	Hydrophilic Area (Å^2)	Hydrophobic Area (Å^2)	Surface (Å^2)
pose 1	114.5	352.4	448.1	310.1	870.1
pose 2	116	402.8	489.3	563.9	1350.9
pose 3	48.9	253.6	302.1	535.9	1031.2
pose 4	69.3	186.5	251.8	425.6	760
pose 5	62.3	304.9	357.7	536.3	1103.7
pose 6	83.4	294.3	373.7	465.7	1025.6
pose 7	90.8	295.4	340.9	693.5	1202.7
average	**83.6**	**298.5**	**370.8**	**502.1**	**1051.2**
st.dv	23.6	63.7	74.7	107.4	184.7

Supplementary Table S2: SiteMap descriptors calculated for ggTas2r1 model2

**MODEL 2**	H-bond acceptor Area (Å^2)	H-bond donor Area (Å^2)	Hydrophilic Area (Å^2)	Hydrophobic Area (Å^2)	Surface (Å^2)
pose 1	53.6	259	305.4	452.6	854.7
pose 2	93.1	332.2	428.3	647.9	1387
pose 3	82.6	215	278.3	400.1	713.1
pose 4	84.4	161.6	253	453.8	1001.2
pose 5	58.2	256.2	314	476.7	978.7
average	**74.4**	**244.8**	**315.8**	**486.2**	**986.9**
st.dv.	15.6	56.2	60.2	84.6	224.8

Supplementary Table 3: Name and SMILES codes of small molecules analyzed by both computational and experimental investigations in this paper

**Name**	**SMILES**
quinine	COc(c1)ccc(c12)nccc2[C@@H](O)[C@H](C[C@@H]34)[N@H+](CC3)C[C@@H]4C=C
diphenhydramine	c1ccccc1C(OCC[NH+](C)C)c2ccccc2
chlorpheniramine	c1cc(Cl)ccc1[C@H](CC[NH+](C)C)c2ncccc2
diphenidol	c1ccccc1C(O)(c2ccccc2)CCC[NH+]3CCCCC3
chloramphenicol	ClC(Cl)C(=O)N[C@H](CO)[C@H](O)c1ccc([NH+]([O-])O)cc1
chloroquine	c1cc(Cl)cc(c12)nccc2N[C@@H](C)CCC[NH+](CC)CC
coumarin	c1cc(=O)oc(c12)cccc2
(8α,9S)-6′-Methoxycinchonan-9-amine trihydrochloride	COc(c1)ccc(c12)nccc2[C@H](N)[C@@H]([N@@H+]34)C[C@@H](CC4)[C@H](C3)C=C
epiquinidine	C=C[C@@H](C[N@H+]12)[C@@H](CC1)C[C@@H]2[C@H](O)c(ccn3)c(c34)cc(cc4)OC
ethylhydrocupreine	CCOc(cc1)cc(c12)c(ccn2)[C@@H](O)[C@H](C[C@H]34)[N@@H+](CC3)C[C@@H]4CC
quinidine	C=C[C@@H](C[N@@H+]12)[C@H](CC1)C[C@@H]2[C@@H](O)c(ccn3)c(c34)cc(cc4)OC

Supplementary Table 4: Receptor activating concentrations of the analyzed molecules

Substance	ggTas2r1	K86A	F89A	N93A	F181A	L185A	Y244A	N247A	L251A
quinine	10	10	3	30	30	30	10	10	10
diphenhydramine	10	10	-	-	-	-	-	-	-
diphenidol	10	30	100	-	30	30	30	30	30
chlorpheniramine	10	10	100	100	10	30	30	30	30
chloramphenicol	10	30	100	30	10	10	10	10	10
chloroquine	10	30	300	100	100	10	30	30	30
coumarin	-	300	-	-	-	-	300	-	-
quinidine	1								
epiquinidine	30								
ethylhydrocupreine	30								
(8α,9S)-6′-Methoxycinchonan-9-amine trihydrochloride	-								

all lowest receptor activating concentrations (=thresholds) in µM

Supplementary Appendix 1: Analysis of ggTas2r1 binding site obtained by modeling the structure using the newly crystallized μ-opioid (5C1M.pdb) and adenosine A2A (5G53.pdb) receptors as templates

Newly crystallized structures in full active conformation, such as adenosine A2A (5G53.pdb) and μ-opioid receptor (5C1M.pdb), were released during the course of this study (on August 2016 and August 2015, respectively).

Below we report the sequence similarity of the binding site of ggTas2r1 compared with the templates used in this work (4MQS.pdb and 2SN6.pdb) and the newly released X-ray structures (5G53.pdb and 5CIM.pdb).

**ggTas2r1**	FKFINSSKRILLCGITTYNFLEVVA	100%
4MQS	YLDYSNWYIFFFGIAYAWYNVWYCY	20%
3SN6	GTDVVTSDFFTYASSSFWFFNYNGY	24%
5G53	AAVLTQGLFEDYFFAYAWLHNMISH	12%
5CIM	QIDYMFGFSPTICIFYAWIHVVFAL	16%

As we may see from the figure above, the similarity of ggTas2r1 with all available templates is very low. The SiteMap descriptors of the binding pockets obtained with 5G53-based and 5CIM-based models are reported below:

	5G53-based model	5CIM-based model
H-bond acceptor Area (Å^2)	133.5	160.7
H-bond donor Area (Å^2)	244.2	175.2
Hydrophilic Area (Å^2)	343.0	319.4
Hydrophobic Area (Å^2)	377.2	407.8
Surface (Å^2)	1050.6	1038.6
